# Sirtuin 2 regulates NOD‐like receptor protein 3/nuclear factor kappa B axis to promote cartilage repair in osteoarthritis

**DOI:** 10.1002/ccs3.70031

**Published:** 2025-07-03

**Authors:** Xiaotian Chen, Yining Song, Fan Zhang, Fangyan Hu, Zhenfei Ding, Jianzhong Guan

**Affiliations:** ^1^ Department of Orthopaedics The First Affiliated Hospital of Bengbu Medical University Bengbu China; ^2^ Anhui Province Key Laboratory of Tissue Transplantation (Bengbu Medical University) Bengbu China; ^3^ School of Pharmacy Bengbu Medical University Bengbu China

**Keywords:** cartilage repair, NF‐κB, NLRP3, osteoarthritis, sirtuin 2

## Abstract

Osteoarthritis (OA) is a prevalent degenerative joint disease driven by inflammation and cartilage degradation. The NOD‐like receptor protein 3 (NLRP3) inflammasome and nuclear factor kappa B (NF‐κB) pathway are central to OA‐associated inflammation. Sirtuin 2 (SIRT2), an NAD^+^‐dependent deacetylase, regulates inflammation and oxidative stress but its role in OA is not fully understood. This study aims to elucidate how SIRT2 modulates the NLRP3/NF‐κB signaling axis to promote cartilage repair in OA. In vivo and in vitro experiments were conducted using OA mouse models and chondrocyte cultures. Single‐cell RNA sequencing was performed to identify differentially expressed genes, followed by Gene Ontology and Kyoto Encyclopedia of Genes and Genomes enrichment analyses. SIRT2's impact on NLRP3 and NF‐κB was assessed using Western blotting (WB), real‐time PCR, co‐immunoprecipitation (Co‐IP), and chromatin immunoprecipitation (ChIP‐qPCR). SIRT2 was found to deacetylate NF‐κB p65, inhibiting NLRP3 activation and reducing inflammatory cytokines. SIRT2 overexpression enhanced chondrocyte proliferation, DNA repair, and mitochondrial function while decreasing reactive oxygen species production. In vivo, SIRT2 significantly improved cartilage repair in OA mice with NLRP3 overexpression attenuating its protective effects. SIRT2 promotes cartilage repair in OA by regulating the NF‐κB/NLRP3 axis, reducing inflammation and oxidative stress. This highlights SIRT2 as a potential therapeutic target for OA.

## Graphical abstract

Schematic diagram illustrating the molecular mechanism by which sirtuin 2 promotes cartilage damage repair in OA through deacetylation, inhibiting the NOD‐like receptor protein 3/nuclear factor kappa B axis.

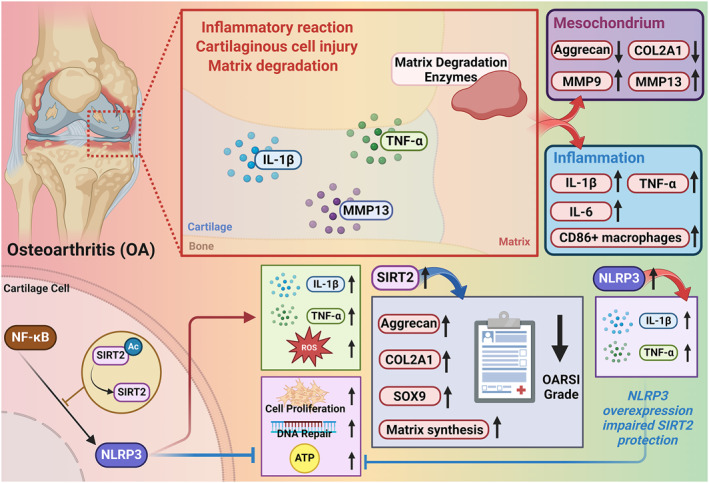

## 1 INTRODUCTION

Osteoarthritis (OA) is a common and prevalent degenerative joint disease characterized primarily by the gradual degeneration and destruction of articular cartilage, accompanied by joint pain and functional impairment.^1^ With the global aging population, the incidence of OA is steadily rising, significantly impacting patients' quality of life.^2^ Current treatments for OA focus primarily on pain management and improving joint function but there is no effective therapy to halt or reverse cartilage damage.^3^ Given the complexity of OA's pathophysiology, particularly the interplay between inflammation and cartilage degeneration, identifying more precise molecular targets has become a central focus of ongoing research.^4^, ^5^

Chronic inflammation is a key factor driving cartilage damage in the progression of OA. The NOD‐like receptor protein 3 (NLRP3) inflammasome and nuclear factor kappa B (NF‐κB) signaling pathways play significant roles in the inflammatory response and cartilage degeneration.^6^, ^7^, ^8^ NLRP3 promotes the expansion of inflammation by inducing the production of pro‐inflammatory cytokines such as interleukin‐1 beta (IL‐1β) and IL‐18, whereas NF‐κB accelerates the degradation of the cartilage matrix by regulating the expression of various inflammatory genes.^9^, ^10^ Therefore, inhibiting the activation of the NLRP3/NF‐κB pathway could effectively reduce inflammation in OA, making it an important target for cartilage repair.

Sirtuins (SIRTs) are a family of NAD^+^‐dependent deacetylases widely distributed in tissues such as the heart, muscle, lung, kidney, brain, and immune system. They play essential roles in various physiological and pathological processes, especially in DNA repair, inflammation regulation, and apoptosis.^11^, ^12^ In addition to the well‐characterized SIRT1, recent studies have also identified the expression of SIRT2, SIRT3, SIRT4, and SIRT6 in cartilage tissue.^13^ SIRT2 is shown to exert anti‐inflammatory effects by modulating the NLRP3 inflammasome and NF‐κB signaling pathways through deacetylation, thereby suppressing the release of pro‐inflammatory cytokines.^14^, ^15^, ^16^ Whereas SIRT2's regulatory role has been demonstrated in various inflammatory and metabolic diseases, its specific mechanism in OA remains to be fully elucidated.^17^, ^18^

Although previous research has highlighted the importance of the NLRP3/NF‐κB signaling pathway in OA‐related inflammation, the molecular mechanism by which SIRT2 regulates this pathway through deacetylation remains unclear.^16^, ^19^, ^20^, ^21^, ^22^ In this study, we used multi‐omics analyses to systematically investigate the role of SIRT2 in cartilage repair during OA, particularly its inhibition of NLRP3 activation through NF‐κB p65 deacetylation. Through in vitro cell experiments and in vivo animal models, we have, for the first time, validated the function of SIRT2 in modulating the NLRP3/NF‐κB axis and comprehensively evaluated its potential therapeutic value in cartilage repair.

The primary objective of this study is to elucidate the molecular mechanism by which SIRT2, through deacetylation of NF‐κB p65, inhibits NLRP3 activation, thereby reducing inflammation and promoting cartilage repair in OA. Through single‐cell RNA sequencing (scRNA‐seq), Western Blot (WB), co‐immunoprecipitation (Co‐IP), chromatin immunoprecipitation‐quantitative polymerase chain reaction (ChIP‐qPCR), and in vivo and in vitro experiments, the regulatory role of SIRT2 was comprehensively evaluated. The results demonstrated that high SIRT2 expression significantly reduced the activation of NLRP3 and NF‐κB, suppressed the secretion of inflammatory factors, and enhanced chondrocyte proliferation and DNA repair capacity. These findings provide novel molecular targets for the diagnosis and treatment of OA and suggest potential therapeutic applications of SIRT2. In the future, the regulatory mechanism of the SIRT2/NF‐κB/NLRP3 axis may offer new directions and possibilities for the precise treatment of OA.

## 2 MATERIALS AND METHODS

### 2.1 Single‐cell RNA sequencing

OA mouse models were constructed (6 mice per group), and cartilage tissues were collected for total RNA extraction using TRIzol reagent (Invitrogen, USA). The RNA concentration and purity were determined using a NanoDrop 2000 spectrophotometer (Thermo Fisher Scientific, USA), and RNA integrity was assessed with a Bioanalyzer 2100 (Agilent Technologies, USA). Samples with an RNA integrity number (RIN) greater than 7 were selected for further experiments.

RNA‐Seq libraries were prepared using the NEBNext Ultra RNA Library Prep Kit for Illumina (NEB, USA). Total RNA was fragmented into approximately 200 bp fragments followed by cDNA synthesis and adapter ligation. Library fragments were purified using AMPure XP Beads (Beckman Coulter, USA) and amplified by PCR. Library quality was evaluated using the Bioanalyzer 2100, and qualified libraries were sequenced on the Illumina HiSeq 4000 platform (Illumina, USA), generating approximately 50 million 100 bp paired‐end reads per sample.

### 2.2 Differential expression analysis

Differential expression analysis was performed using the “limma” package in R software. The *p*‐values for differential expression were adjusted using the false discovery rate (FDR) method. Genes and proteins with significant differential expression were identified based on the thresholds of FDR <0.05 and |log_2_FC| > 1. Volcano plots were generated using the “ggplot2” package in R. Comparisons between the two groups were conducted using the Wilcoxon test. All analyses were performed in R version 4.2.1 (R Foundation for Statistical Computing).

### 2.3 GO and KEGG functional enrichment analysis

Gene Ontology (GO) and Kyoto Encyclopedia of Genes and Genomes (KEGG) enrichment analyses of the differentially expressed genes (DEGs) were conducted using the “ClusterProfiler” package in R. The KEGG enrichment results were visualized with the “ggplot2” package. A threshold of *p* < 0.05 was set to identify significantly enriched cellular functions and signaling pathways among the DEGs.

### 2.4 Gene correlation analysis

Spearman correlation analysis was performed to evaluate the relationship between SIRT2 and the NLRP3/NF‐κB signaling axis in OA. The correlation results were visualized using the “ggplot2” package in R. A positive correlation coefficient indicates a direct relationship between the two variables, whereas a negative correlation coefficient signifies an inverse relationship.

### 2.5 Isolation and culture of mouse chondrocytes

Chondrocytes were isolated from the tibial plateau cartilage of 5‐day‐old C57BL/6 mice using sterile techniques. First, cartilage tissue was cut into small pieces and digested in 0.25% trypsin (25200‐072, Gibco, Thermo Fisher Scientific, USA) at 37°C for 30 min. The tissue was then washed three times with phosphate‐buffered saline (PBS). Following this, the cartilage was further digested in a 0.05% collagenase IV solution (LS004188, Worthington Biochemical Corporation, USA) at 37°C for 6 h. After digestion, the resulting cell suspension was filtered through a 70 μm cell strainer (352350, Falcon, Corning, USA) and centrifuged at 1000 rpm for 5 min to collect the cell pellet. The isolated chondrocytes were resuspended in DMEM medium (11965092, Gibco, USA), supplemented with 10% fetal bovine serum (FBS, 26140079, Gibco, USA) and 100 U/mL penicillin‐streptomycin (15140122, Gibco, USA). The cells were cultured in a cell incubator (model: HERAcell 150i, Thermo Fisher Scientific, USA) at 37°C with 5% CO_2_ until further use. All experiments were performed using P2 (second passage) chondrocytes.

### 2.6 Establishing an OA cell model

To establish an OA cell model, primary chondrocytes were treated with 10 ng/mL of tumor necrosis factor‐alpha (TNF‐α) (TNF‐α, 210‐TA, R&D Systems, USA) for 24 h to induce an inflammatory response, simulating the pathological environment of OA.^23^

### 2.7 Cell transfection and grouping

To investigate the function of SIRT2 in mouse chondrocytes, we employed lentivirus‐mediated shRNA to knock down SIRT2 expression and plasmid‐mediated overexpression for SIRT2 and NF‐κB p65. Lentiviral vectors for sh‐SIRT2 transduction (purchased from GeneChem, China) were used to suppress SIRT2 expression in mouse chondrocytes. The lentivirus was added to the cultured chondrocytes at a multiplicity of infection (MOI) of 50. To enhance transduction efficiency, 5 μg/mL polybrene (Sigma‐Aldrich, USA) was included in the medium. After 24 h, the medium was replaced with fresh DMEM (Gibco, USA), and the cells were cultured for an additional 72 h to ensure stable expression. For the overexpression of SIRT2 and NF‐κB p65, plasmid‐mediated transfection was performed using plasmids for SIRT2 and NF‐κB p65 (both obtained from Addgene, USA). The plasmids were transfected into the chondrocytes using Lipofectamine 3000 (Invitrogen, USA). Specifically, 2.5 μg of plasmid DNA and 5 μL of Lipofectamine 3000 were added to each well of a six‐well plate and incubated in serum‐free Opti‐MEM medium (Gibco, USA) for 20 min before adding to the cells. Six hours post‐transfection, the medium was replaced with DMEM containing 10% fetal bovine serum (FBS, Gibco, USA), and the cells were cultured for an additional 48 h. The experimental groups were as follows: control group: untreated cells. sh‐NC group: cells transduced with lentiviral shRNA empty vector. Model group: cells treated with TNF‐α to simulate an OA model. sh‐SIRT2 group: cells transduced with lentiviral sh‐SIRT2. oe‐SIRT2 group: cells transfected with SIRT2 overexpression plasmid. oe‐SIRT2 + oe‐NF‐κB group: cells co‐transfected with SIRT2 and NF‐κB p65 overexpression plasmids. The sequences of the lentiviral shRNA are listed in Table S1.

### 2.8 CCK‐8 assay

To evaluate the effects of SIRT2 and NF‐κB p65 on the proliferation of mouse chondrocytes, cell viability was assessed using the cell counting kit‐8 (CCK‐8) assay kit (Dojindo, Japan). Transfected cells were seeded in 96‐well plates at a density of 1 × 10^4^ cells per well with six replicates for each group. After 24 h of incubation, 10 μL of CCK‐8 solution was added to each well followed by an additional 2‐h incubation. The absorbance was measured at 450 nm using a microplate reader (Thermo Fisher Scientific, USA).

### 2.9 WB analysis

Total proteins were extracted from the cells or tissues using RIPA lysis buffer (Thermo Fisher Scientific, USA), and protein concentrations were determined using a BCA protein assay kit (Thermo Fisher Scientific, USA). For each sample, 30 μg of protein was loaded onto SDS‐PAGE gels for electrophoresis followed by transfer to PVDF membranes (Millipore, USA). The membranes were incubated with the following primary antibodies: anti‐SIRT2 (ab211033, 1:1000, Abcam, UK), anti‐NLRP3 (15101, 1:1000, Cell Signaling Technology, USA), anti‐NF‐κB p65 (8242, 1:1000, Cell Signaling Technology, USA), anti‐IL‐1β (ab315084, 1:1000, Abcam, UK), anti‐COL2A1 (ab34712, 1:2000, Abcam, UK), anti‐aggrecan (ab313636, 1:1000, Abcam, UK), anti‐MMP9 (24317, 1:1000, Cell Signaling Technology, USA), anti‐MMP13 (67329, 1:1000, Cell Signaling Technology, USA), and GAPDH (ab8245, 1:5000, Abcam, UK). Secondary antibodies, HRP‐conjugated anti‐rabbit (ab6721, 1:5000, Abcam, UK) or anti‐mouse (ab6789, 1:5000, Abcam, UK), were used for detection. Protein bands were visualized using the ECL detection kit (Thermo Fisher Scientific, USA) and imaged with the Bio‐Rad imaging system.

### 2.10 Real‐time quantitative polymerase chain reaction (RT‐qPCR) analysis

Total RNA was extracted from the target cells using TRIzol reagent (Thermo Fisher Scientific, USA) and reverse transcribed into cDNA with the PrimeScript RT kit (Takara, Japan). Quantitative PCR was then performed using SYBR Green PCR master mix (Thermo Fisher Scientific, USA) on an ABI 7500 real‐time PCR system (Applied Biosystems, USA). Relative gene expression levels were calculated using the ΔΔCt method. The target genes included NLRP3, SIRT2, NF‐κB p65, and IL‐1β (primer sequences are provided in Table S2) with GAPDH as the internal control. Each sample was tested in triplicate.

### 2.11 Co‐IP

To examine the interaction between SIRT2 and NF‐κB p65, mouse chondrocytes were lysed following transfection with FLAG‐SIRT2 and Myc‐NF‐κB p65 plasmids. The lysis buffer contained protease inhibitors (Roche, Switzerland) and RIPA lysis buffer (Thermo Fisher Scientific, USA). After lysis, the cell lysates were centrifuged to collect the supernatant followed by protein quantification. The lysates were incubated overnight at 4°C with 1 μg of either anti‐FLAG antibody (F1804, Sigma‐Aldrich, USA; dilution: 1:200) or anti‐Myc antibody (sc‐40, Santa Cruz Biotechnology, USA; dilution: 1:200). Subsequently, protein A/G magnetic beads (Thermo Fisher Scientific, USA) were added and incubated with gentle rotation for 2 h at 4°C to capture the antigen‐antibody complexes. The beads were then washed three times with RIPA buffer, each wash lasting 5 min. The immunoprecipitated complexes were dissociated by boiling at 95°C for 5 min. The samples were separated by SDS‐PAGE and transferred to a PVDF membrane (Millipore, USA). NF‐κB p65 protein was detected using an anti‐NF‐κB p65 antibody (8242, Cell Signaling Technology, USA; dilution: 1:1000). Protein signals were visualized using ECL detection (Thermo Fisher Scientific, USA).

### 2.12 Detection of deacetylation levels

To assess the deacetylation modification of NF‐κB p65 by SIRT2, cells were transfected with the Myc‐NF‐κB p65 plasmid followed by immunoprecipitation using an anti‐Myc antibody. The acetylation level of NF‐κB p65 was then detected using an anti‐acetylated lysine antibody (Cell Signaling Technology, USA). GAPDH was used as a loading control, and protein levels were analyzed by WB.

### 2.13 Dual‐luciferase reporter assay

To evaluate the effect of NF‐κB on NLRP3 promoter activity, the NLRP3 promoter region was first cloned into the pGL3‐basic reporter plasmid (Promega, USA) and co‐transfected with a Renilla luciferase control plasmid into the target cells. After 24 h of TNF‐α treatment, luciferase activity was measured using the dual‐luciferase reporter assay system (Promega, USA). The experiments included individual and combined overexpression of SIRT2 and NF‐κB p65 to assess their effects on NLRP3 promoter activity. The results were presented as the ratio of firefly luciferase activity to Renilla luciferase activity.

### 2.14 ChIP‐qPCR assay

To validate the binding of NF‐κB p65 to the NLRP3 promoter region, we performed a chromatin immunoprecipitation (ChIP) assay. First, treated cells were cross‐linked with 1% formaldehyde to fix DNA‐protein interactions. Chromatin was then fragmented using sonication. Immunoprecipitation was carried out using an anti‐NF‐κB p65 antibody (Santa Cruz Biotechnology, USA) and protein A/G magnetic beads (Thermo Fisher Scientific, USA). After extracting the immunoprecipitated DNA, quantitative polymerase chain reaction (qPCR) was performed using SYBR Green PCR master mix on the ABI 7500 real‐time PCR system to assess the enrichment of the NLRP3 promoter region. The GAPDH promoter was used as a negative control.

### 2.15 γ‐H2AX immunofluorescence staining

To detect DNA damage in chondrocytes, γ‐H2AX immunofluorescence staining was performed. After treatment, the cells were fixed with 4% paraformaldehyde (Sigma‐Aldrich, USA) for 15 min and permeabilized with 0.1% Triton X‐100 (Sigma‐Aldrich, USA) for 10 min. Next, the cells were incubated overnight at 4°C with an anti‐γ‐H2AX antibody (ab81299, 1:250, Abcam, UK). On the following day, the cells were washed with PBS and incubated with a secondary antibody, goat anti‐rabbit IgG H&L (Alexa Fluor® 555) (ab150078, 1:1000, Abcam, UK), at room temperature for 1 h. Finally, the cells were visualized and imaged using a fluorescence microscope (Leica Microsystems, Germany). DAPI (Sigma‐Aldrich, USA) was used for nuclear staining.

### 2.16 Cell cycle analysis

Cell cycle analysis was performed using flow cytometry (FCM). After transfection, the treated cells were collected using PBS and fixed in 70% ethanol at 4°C overnight. The cells were then stained for 30 min with a staining solution containing 50 μg/mL propidium iodide (PI) (PI, Sigma‐Aldrich, USA) and RNase A (Thermo Fisher Scientific, USA). PI fluorescence signals were detected using a flow cytometer (BD Biosciences, USA), and cell cycle distribution was analyzed using ModFit LT software.

### 2.17 Apoptosis detection

Apoptosis was detected using an Annexin V‐FITC/PI double staining kit (BD Biosciences, USA). After treatment, the cells were washed with PBS and resuspended in a binding buffer. Subsequently, 5 μL of Annexin V‐FITC and 5 μL of PI were added, and the cells were incubated in the dark for 15 min. FCM (BD Biosciences, USA) was used for analysis, and the percentage of apoptotic cells was quantified using FlowJo software.

### 2.18 ROS level detection

To assess intracellular reactive oxygen species (ROS) levels, the 2′,7′‐dichlorofluorescein diacetate (DCFH‐DA) fluorescent probe (Sigma‐Aldrich, USA) was used. The treated cells were incubated with 10 μM DCFH‐DA for 30 min followed by washing with PBS. ROS fluorescence signals were observed using a fluorescence microscope (Leica Microsystems, Germany). The increase in fluorescence intensity was used to evaluate ROS levels. Additionally, quantitative detection of ROS production was performed using a ROS enzyme‐linked immunosorbent assay (ELISA) kit (E‐BC‐F005, Elabscience, Wuhan, China) following the manufacturer's instructions. Absorbance was measured at a wavelength of 450 nm using a microplate reader.

### 2.19 Immunofluorescence staining of F‐actin and β‐tubulin

Immunofluorescence staining was employed to observe the distribution of F‐actin and β‐tubulin. First, treated cells were fixed with 4% paraformaldehyde (Sigma‐Aldrich, USA) for 15 min followed by permeabilization with 0.1% Triton X‐100 (Sigma‐Aldrich, USA) for 10 min. After blocking the cells with 1% BSA in PBS for 30 min, primary antibodies against F‐actin (ab205, 1:200, Abcam, UK) and β‐tubulin (ab18207, 1:50, Abcam, UK) were added, and the cells were incubated overnight at 4°C. The next day, cells were washed with PBS and incubated for 1 h at room temperature in the dark with Alexa Fluor 488 (ab150077, 1:100, Abcam, UK) or Alexa Fluor 594 (ab150080, 1:100, Abcam, UK)‐conjugated secondary antibodies. DAPI (Sigma‐Aldrich, USA) was used to stain the nuclei. The distribution and morphology of F‐actin and β‐tubulin were observed using a fluorescence microscope (Leica Microsystems, Germany).

### 2.20 ATP level measurement

The intracellular adenosine triphosphate (ATP) levels were quantified using an ATP assay kit (E‐BC‐F002, Elabscience, Wuhan, China). Treated cells were seeded into 96‐well plates at a density of 1 × 10^4^ cells per well. After treatment, ATP lysis buffer was added according to the manufacturer's instructions. Absorbance was then measured at 570 nm using a microplate reader (Thermo Fisher Scientific, USA).

### 2.21 TEM analysis of mitochondrial morphology

To observe changes in mitochondrial quantity and morphology, a transmission electron microscope (TEM, JEOL, Japan) was used. Treated chondrocytes were fixed with 2.5% glutaraldehyde (Sigma‐Aldrich, USA) for 24 h followed by a graded ethanol dehydration process and embedding in epoxy resin. Ultrathin sections were prepared and double‐stained with 2% uranyl acetate and lead citrate (both from Thermo Fisher Scientific, USA). Mitochondrial quantity, morphology, and ultrastructure were then examined using TEM.

### 2.22 Establishment of the OA mouse model

To establish an OA mouse model, the destabilization of the medial meniscus (DMM) method was used to induce OA. Eight‐week‐old male C57BL/6 mice (strain code: 219) were purchased from Beijing Vital River Laboratory Animal Technology Co., China. The surgical procedure involved the following steps: after administering general anesthesia using isoflurane (RWD Life Science, China), the knee joint was exposed, and the attachment of the medial meniscus to the tibia was severed, causing joint instability. In the sham group, only the joint capsule was incised without severing the meniscal attachment. After surgery, the mice were randomly divided into the following experimental groups: control group, DMM group (OA model), SIRT2 overexpression group, SIRT2 knockdown group, and rescue group (co‐overexpression of SIRT2 and NLRP3). Three weeks post‐surgery, SIRT2 overexpression and knockdown were achieved by delivering SIRT2 plasmids or shRNA via an adeno‐associated virus (AAV) vector with injections into the joint cavity twice a week for 3 weeks. In the rescue group, NLRP3 overexpression plasmids were co‐injected alongside SIRT2 overexpression. One week after the final injection (week 7 post‐DMM surgery), mouse samples were collected for further experimental analysis.^23^

### 2.23 OARSI pathological scoring

To assess the degree of cartilage damage, the osteoarthritis research society international (OARSI) scoring system was used. Mouse knee joints were collected and fixed in 10% formalin for 24 h, followed by decalcification, paraffin embedding, and sectioning into consecutive slices (5 μm). The sections were stained with hematoxylin and eosin (H&E) and safranin O to evaluate cartilage damage. Two independent observers scored the articular cartilage using the OARSI scale, ranging from 0 (normal) to 6 (severe damage), and the average score was recorded.

### 2.24 IHC analysis

Immunohistochemistry (IHC) analysis was performed to detect the expression of cartilage repair markers (COL2A1, Aggrecan, and SOX9). Paraffin‐embedded knee joint sections were deparaffinized, rehydrated, and subjected to antigen retrieval in citrate buffer (pH 6.0) at 95°C. Endogenous peroxidase activity was blocked using 3% hydrogen peroxide solution. The sections were then incubated overnight with primary antibodies: anti‐COL2A1 (ab34712, 1:200, Abcam, UK), anti‐Aggrecan (ab313636, 1:500, Abcam, UK), and anti‐SOX9 (ab185966, 1:500, Abcam, UK). On the following day, the sections were incubated with HRP‐conjugated secondary antibodies followed by color development using DAB (Thermo Fisher Scientific, USA). Hematoxylin was used for nuclear counterstaining. The results were observed under a microscope (Leica Microsystems, Germany), and the percentage of positively stained areas was quantified using ImageJ software.

### 2.25 FCM analysis

To assess inflammatory cell infiltration, immune cells were first isolated from joint tissues. Periarticular tissue from the knee joints was collected and subjected to mechanical dissociation followed by enzymatic digestion using Collagenase I/II (Sigma‐Aldrich, USA). The resulting cell suspension was filtered and washed with PBS. Cells were then stained with antibodies against CD45, CD11b, and CD86 (all from BD Biosciences, USA) and incubated in the dark at 4°C for 30 min. The proportions of CD45^+^ leukocytes, CD11b^+^ macrophages, and CD86^+^ activated macrophages were subsequently analyzed using a flow cytometer (BD Biosciences, USA). Data analysis was performed using FlowJo software.

### 2.26 ELISA assay

The levels of inflammatory cytokines IL‐1β (MLB00C, R&D Systems, USA), TNF‐α (MTA00B, R&D Systems, USA), and IL‐6 (M6000B, R&D Systems, USA) were measured using ELISA kits. Joint synovial fluid and serum were collected from mice for analysis. The assay was performed according to the manufacturer's instructions, with samples incubated with specific antibodies, followed by incubation with HRP‐conjugated secondary antibodies for color development. Absorbance was measured at 450 nm using a microplate reader (Thermo Fisher Scientific, USA). A standard curve was generated for each experiment to quantify the concentrations of the inflammatory cytokines.

### 2.27 Statistical analysis

All experimental data are presented as the mean ± standard deviation (SD). Statistical analyses were performed using GraphPad Prism 8.0 software (GraphPad Software, USA). Comparisons between groups were conducted using analysis of variance (ANOVA) followed by Tukey's HSD test. For pairwise comparisons, two‐tailed *t*‐tests or ANOVA were applied, and for non‐normally distributed data, the Mann–Whitney *U* test was used. Statistical significance was set at *p* < 0.05.

## 3 RESULTS

### 3.1 scRNA‐seq reveals that SIRT2 inhibits the NLRP3/NF‐κB pathway through deacetylation

**FIGURE 1:**
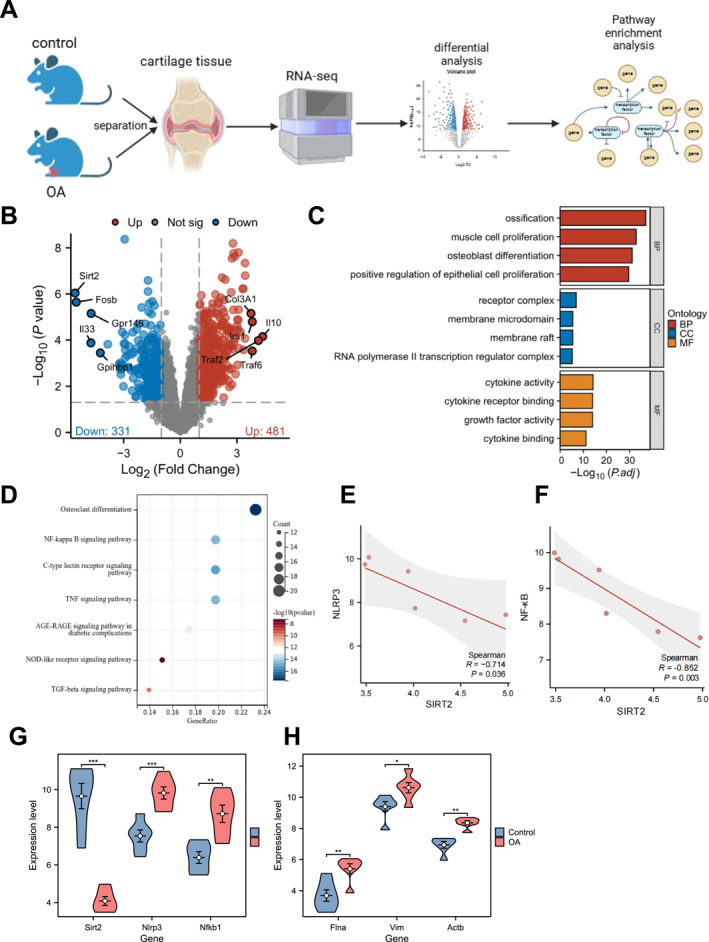
Mechanistic analysis of how SIRT2 inhibits the NLRP3/NF‐κB signaling pathway through deacetylation. (A) Schematic diagram of the single‐cell RNA sequencing workflow. (B) Volcano plot of differential gene expression with red dots indicating upregulated genes in OA cartilage tissue, blue dots indicating downregulated genes, and gray dots representing genes with no significant differences. (C) Gene Ontology enrichment analysis showing the biological processes significantly enriched for DEGs with the *x*‐axis representing the enrichment level and the *y*‐axis representing biological processes. (D) The circular plot of Kyoto Encyclopedia of Genes and Genomes pathway enrichment analysis, highlighting the pathways significantly enriched for DEGs. (E) Correlation analysis between SIRT2 and NLRP3 expression. (F) Correlation analysis between SIRT2 and NF‐κB expression. (G) Bar graph showing the expression levels of Sirt2, Nlrp3, and Nfkb1 in OA. (H) Bar graph showing the expression of Flna, Vim, and Actb in OA cartilage tissue. DEGs, differentially expressed genes; NF‐κB, nuclear factor kappa B; NLRP3, NOD‐like receptor protein 3; SIRT2, sirtuin 2.

In this study, we performed scRNA‐seq on cartilage tissue from both normal mice and OA mouse models (Figure 1A), identifying 812 significant DEGs, with 481 genes upregulated and 331 genes downregulated (Figure 1B). Among these, Traf2, Traf6, and Sirt2 were highlighted with Sirt2 showing the most significant downregulation. To further investigate the biological functions of these genes, we conducted GO and KEGG enrichment analyses. GO analysis revealed significant enrichment of these genes in bone‐related biological processes such as “ossification,” “osteoblast differentiation,” and “myoblast proliferation” as well as in molecular functions associated with inflammatory responses including “cytokine activity” and “cytokine receptor binding” (Figure 1C). KEGG analysis identified the NF‐κB signaling pathway as a key pathway involving these genes, suggesting that inflammation plays a central role in the pathogenesis of OA (Figure 1D). Correlation analysis demonstrated a significant negative relationship between SIRT2 expression and the NLRP3/NF‐κB signaling axis (Figure 1E,F). In OA, SIRT2 expression was significantly downregulated, whereas NLRP3 and NF‐κB expression levels were markedly elevated (Figure 1G), suggesting that SIRT2 may modulate inflammation and DNA damage repair in OA by suppressing this signaling cascade. Moreover, differential gene expression analysis showed that several cytoskeleton‐related proteins, including Flna, Vim, and Actb, were significantly upregulated in OA cartilage tissue (Figure 1H), indicating that cytoskeletal remodeling may play a key role in chondrocyte dysfunction and OA pathogenesis. Based on literature reports, SIRT2 has been shown to regulate biological functions through deacetylation, particularly by deacetylating histone H4K16.^24^ Thus, we hypothesize that SIRT2 exerts its anti‐inflammatory effects by deacetylating H4K16, thereby regulating the NLRP3/NF‐κB axis, making it a potential therapeutic target for OA.

### 3.2 SIRT2 inhibits NF‐κB expression through deacetylation modification

**FIGURE 2:**
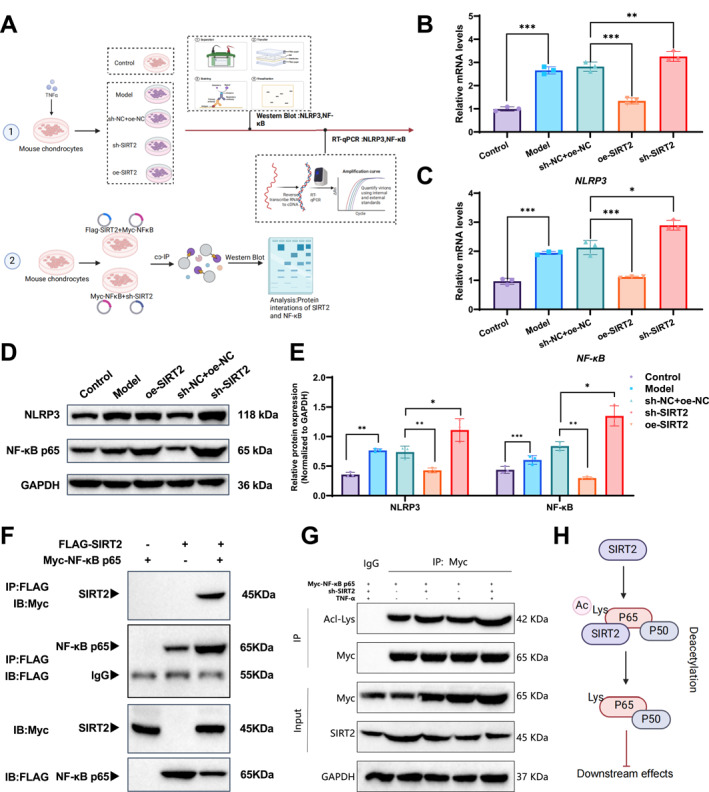
The regulatory role of SIRT2 on the NF‐κB pathway in mouse chondrocytes and validation of its deacetylation modifications. (A) Schematic of the experimental design. (B) RT‐qPCR showing effects of SIRT2 overexpression and knockdown on NLRP3 mRNA expression. (C) RT‐qPCR showing effects on NF‐κB mRNA expression. (D) Western blot results demonstrating protein expression changes of NLRP3 and NF‐κB. (E) Quantification of NLRP3 and NF‐κB protein levels across groups. (F) Co‐IP experiment demonstrating the interaction between SIRT2 and NF‐κB p65. (G) Co‐IP experiment showing the acetylation level of NF‐κB p65 under SIRT2 knockdown conditions. (H) Schematic illustrating the mechanism by which SIRT2 deacetylates NF‐κB p65, inhibiting its acetylation. All data are presented as mean ± standard error of the mean with experiments repeated three times. Statistical analyses were conducted using analysis of variance with Tukey's post hoc test. *p < 0.05, **p < 0.01, ***p < 0.001. NF‐κB, nuclear factor kappa B; NLRP3, NOD‐like receptor protein 3; RT‐qPCR, real‐time quantitative polymerase chain reaction; SIRT2, sirtuin 2.

Previous omics analyses revealed a significant negative correlation between SIRT2 and the NLRP3/NF‐κB axis. To validate these findings, we treated murine chondrocytes with TNF‐α to simulate an OA cellular model (Figure 2A) and analyzed the changes in NLRP3 and NF‐κB expression following SIRT2 overexpression and knockdown using WB and RT‐qPCR. Western blot and RT‐qPCR were used to evaluate the expression changes of NLRP3 and NF‐κB after SIRT2 overexpression or knockdown. We first confirmed the efficacy of SIRT2 modulation: compared to the sh‐NC group, SIRT2 knockdown (sh‐SIRT2) significantly reduced SIRT2 mRNA and protein levels in chondrocytes (Figure S1A,B), whereas SIRT2 overexpression (oe‐SIRT2) markedly increased both mRNA and protein levels (Figure S1C,D). Functionally, the results demonstrated that overexpression of SIRT2 significantly reduced the mRNA and protein levels of NLRP3 and NF‐κB while SIRT2 knockdown led to a marked increase in the expression of these molecules (Figure 2B–E). Additionally, Co‐IP assays further confirmed the interaction between SIRT2 and NF‐κB p65 (Figure 2F). When FLAG‐tagged SIRT2 was overexpressed, Myc‐tagged NF‐κB p65 was co‐precipitated, indicating that SIRT2 regulates NF‐κB p65. To further investigate the impact of SIRT2 on NF‐κB p65 deacetylation, we conducted immunoprecipitation using a Myc antibody in cells transfected with Myc‐tagged NF‐κB p65 plasmids and knocked down SIRT2. To further assess whether SIRT2 regulates NF‐κB p65 acetylation and whether this regulation is influenced by TNF‐α, we performed IP with anti‐Myc antibody in different treatment groups transfected with Myc‐tagged NF‐κB p65 plasmid. The acetylation level of NF‐κB p65 was then detected using an anti‐acetylated lysine antibody. The results showed that both TNF‐α stimulation alone and SIRT2 knockdown significantly increased NF‐κB p65 acetylation compared to the control group. Moreover, SIRT2 knockdown further enhanced p65 acetylation beyond that induced by TNF‐α alone. The input confirmed that Myc‐NF‐κB p65 and SIRT2 were expressed consistently across all samples with GAPDH as a loading control showing no significant changes (Figure 2G). These findings suggest that SIRT2 deacetylates NF‐κB p65, inhibiting its acetylation modification (Figure 2H).

### 3.3 SIRT2 inhibits NLRP3 activation by regulating NF‐κB

**FIGURE 3:**
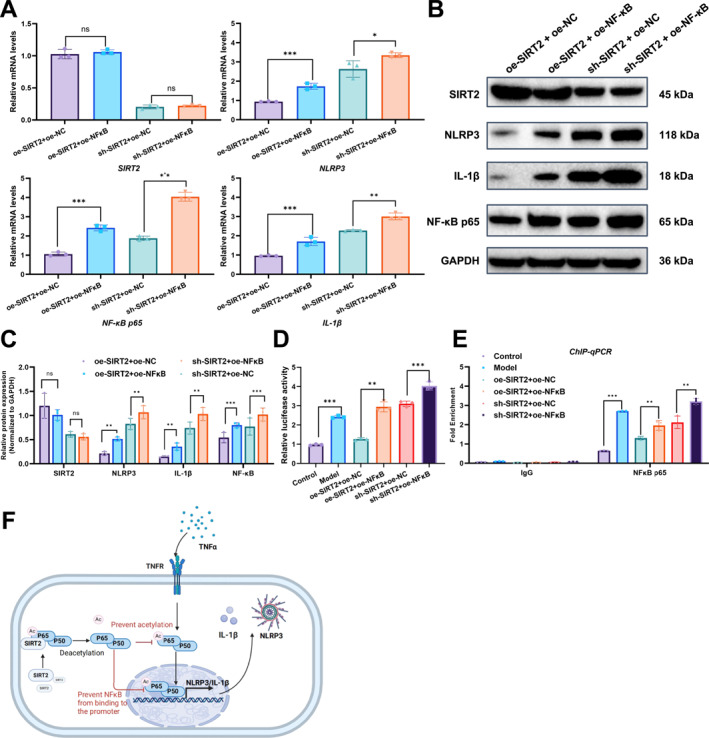
SIRT2 inhibits NLRP3 activation by regulating NF‐κB p65 activity. (A–C) Changes in mRNA and protein expression levels of NLRP3 and IL‐1β under various conditions, including SIRT2 overexpression or knockdown, as well as the co‐transfection effect of NF‐κB p65. (D) Dual‐luciferase reporter assay showing NF‐κB binding activity to the NLRP3 promoter under TNF‐α treatment and the effect of SIRT2 and NF‐κB p65 overexpression. (E) ChIP‐qPCR assay detecting NF‐κB p65 enrichment at the NLRP3 promoter region and evaluating the impact of SIRT2 and NF‐κB p65 overexpression on this binding. (F) Schematic diagram illustrating the mechanism by which SIRT2 inhibits NLRP3 activation through suppression of NF‐κB p65 activity, reducing its binding to the NLRP3 promoter region. All data are presented as mean ± standard error of the mean, with experiments repeated three times. Statistical analysis was performed using analysis of variance with Tukey's post‐hoc test. NF‐κB, nuclear factor kappa B; NLRP3, NOD‐like receptor protein 3; SIRT2, sirtuin 2. *p < 0.05, **p < 0.01, ***p < 0.001.

In previous experiments, we observed that the overexpression or knockdown of SIRT2 significantly affected NLRP3 expression levels. To further investigate whether SIRT2 inhibits NLRP3 activation through the suppression of NF‐κB, we co‐transfected SIRT2 and NF‐κB p65 overexpression plasmids and measured the changes in NLRP3 and IL‐1β expression. The results showed that under SIRT2 overexpression, the levels of NLRP3 and IL‐1β were significantly reduced. However, this inhibitory effect was partially reversed when NF‐κB p65 was co‐overexpressed. Conversely, in SIRT2 knockdown conditions, NLRP3 and IL‐1β expression levels were markedly increased, and NF‐κB p65 overexpression further amplified this effect (Figure 3A–C).

Dual‐luciferase reporter assays demonstrated that NF‐κB was significantly activated and enhanced its binding to the NLRP3 promoter under TNF‐α treatment. SIRT2 overexpression partially suppressed this activation; however, the inhibitory effect was partially reversed when NF‐κB p65 was overexpressed (Figure 3D). To further validate SIRT2's regulatory effect on NF‐κB target genes, we performed ChIP‐qPCR to assess the enrichment of NF‐κB p65 at the NLRP3 promoter region. The results revealed that TNF‐α treatment significantly increased NF‐κB p65 binding to the NLRP3 promoter. SIRT2 overexpression partially inhibited this binding, suggesting a negative regulatory role for SIRT2 on NF‐κB p65 activity. However, this inhibition was also partially reversed under NF‐κB p65 overexpression (Figure 3E). Together, the dual‐luciferase and ChIP‐qPCR results indicate that SIRT2 suppresses NLRP3 activation by modulating NF‐κB p65 binding activity.

In conclusion, these findings suggest that SIRT2 inhibits NLRP3 activation by reducing NF‐κB p65 binding to the NLRP3 promoter (Figure 3F), further highlighting the critical role of SIRT2 in inflammation regulation.

### 3.4 SIRT2 inhibits chondrocyte damage via the NF‐κB/NLRP3 axis

**FIGURE 4:**
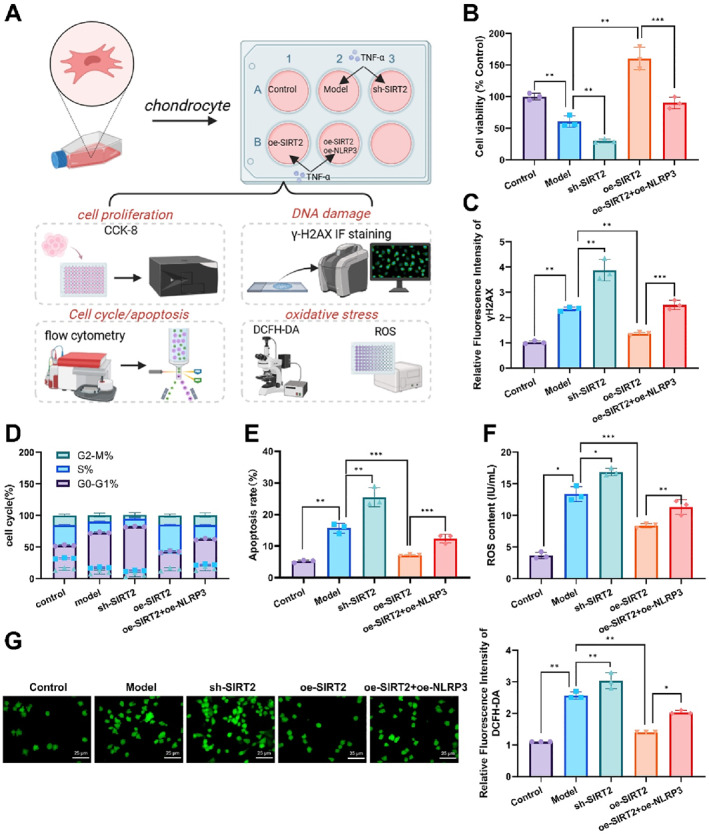
In vitro results of SIRT2 regulating chondrocyte damage via the NF‐κB/NLRP3 axis. (A) Schematic diagram of the in vitro experimental process. (B) CCK8 assay evaluating chondrocyte proliferation across different groups. (C) γ‐H2AX immunofluorescence staining indicating DNA damage marker expression in each group of chondrocytes. (D) Cell cycle analysis showing the distribution of cells in the G1 and S phases for each group. (E) Apoptosis rates of chondrocytes in each group measured by FCM. (F–G) enzyme‐linked immunosorbent assay and DCFH‐DA fluorescence staining assessing reactive oxygen species production in chondrocytes across different groups. Data are presented as mean ± standard error of the mean with *p < 0.05, **p < 0.01, and ***p < 0.001. NF‐κB, nuclear factor kappa B; NLRP3, NOD‐like receptor protein 3; SIRT2, sirtuin 2.

In this study, we investigated the effect of SIRT2 on chondrocyte damage by regulating the NF‐κB/NLRP3 axis through in vitro experiments as outlined in Figure 4A. CCK8 assays demonstrated that SIRT2 overexpression significantly promoted chondrocyte proliferation, whereas SIRT2 knockdown markedly inhibited cell proliferation. Moreover, co‐overexpression of SIRT2 and NLRP3 partially reversed the pro‐proliferative effect of SIRT2 overexpression (Figure 4B). γ‐H2AX staining revealed that SIRT2 knockdown led to a notable increase in the fluorescence signal of the DNA damage marker γ‐H2AX, indicating exacerbated cellular damage, whereas SIRT2 overexpression reduced the γ‐H2AX signal. Notably, the DNA damage marker signal in the oe‐SIRT2+oe‐NLRP3 group was higher than that in the SIRT2 overexpression group, suggesting that NLRP3 overexpression mitigated the protective effect of SIRT2 (Figure 4C).

Cell cycle analysis (Figure 4D, Figure S2A) showed that SIRT2 overexpression promoted the progression from G1 to S phase while SIRT2 knockdown caused G1 phase arrest. The cell cycle progression in the oe‐SIRT2+oe‐NLRP3 group was intermediate between the SIRT2 overexpression and knockdown groups, indicating that NLRP3 overexpression partially hindered SIRT2's regulation of the cell cycle. Apoptosis analysis (Figure 4E, Figure S2B) revealed that SIRT2 knockdown significantly increased the apoptosis rate while SIRT2 overexpression reduced it. The apoptosis rate in the oe‐SIRT2+oe‐NLRP3 group was higher than in the SIRT2 overexpression group, indicating that NLRP3 overexpression promoted apoptosis.

In the ROS detection assays, ELISA and DCFH‐DA fluorescence staining demonstrated that SIRT2 overexpression significantly reduced ROS levels, whereas SIRT2 knockdown increased ROS production. In the oe‐SIRT2+oe‐NLRP3 group, ROS levels were higher than in the SIRT2 overexpression group, suggesting that NLRP3 overexpression partially counteracted SIRT2's inhibitory effect on ROS production (Figure 4F,G). These results indicate that SIRT2 plays a crucial role in regulating chondrocyte damage, proliferation, apoptosis, and ROS generation through the NF‐κB/NLRP3 axis and that NLRP3 overexpression can attenuate some of SIRT2's protective effects.

### 3.5 SIRT2 promotes cartilage repair by regulating cytoskeletal reorganization and energy metabolism via the NF‐κB/NLRP3 axis in chondrocytes

**FIGURE 5:**
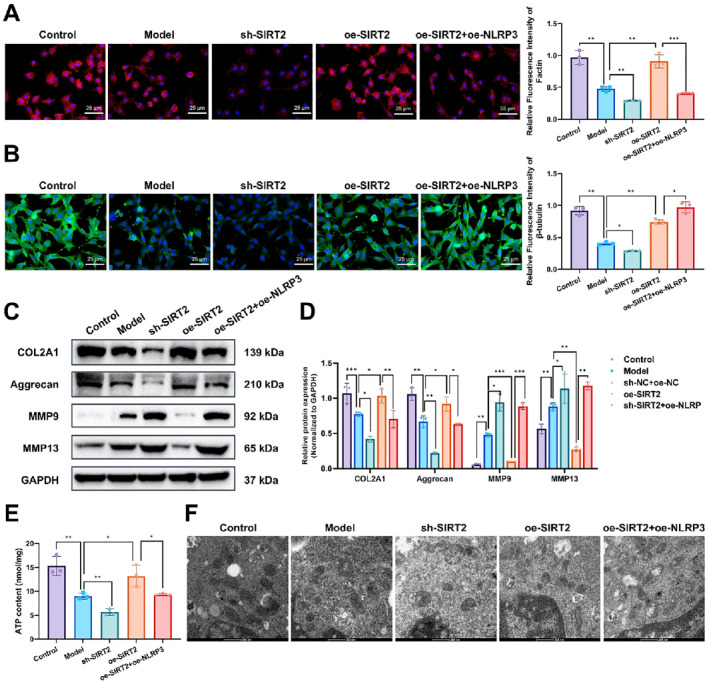
The effects of SIRT2 on cytoskeletal reorganization and energy metabolism in chondrocytes via the NF‐κB/NLRP3 axis. (A, B) Immunofluorescence staining showing the distribution of F‐actin and β‐tubulin in chondrocytes from each group. (C, D) WB analysis of cartilage repair‐related proteins, including COL2A1, aggrecan, and matrix‐degrading enzymes matrix metalloproteinase 9 and MMP13, across different groups. (E) ATP levels in chondrocytes from each group. (F) Electron microscopy images displaying the number and morphology of mitochondria in chondrocytes from each group. *Data are presented as mean ± standard error of the mean with *p < 0.05, **p < 0.01, and ***p < 0.001. NF‐κB, nuclear factor kappa B; NLRP3, NOD‐like receptor protein 3; SIRT2, sirtuin 2.

We further investigated the role of SIRT2 in regulating the NF‐κB/NLRP3 axis and its effects on cytoskeletal reorganization and energy metabolism in chondrocytes. Immunofluorescence staining revealed that in the control group, F‐actin and β‐tubulin were evenly distributed in chondrocytes, whereas in the model group, the distribution of both cytoskeletal proteins was significantly reduced, indicating a disruption in cytoskeletal structure. In contrast, the SIRT2 overexpression group showed a marked increase in F‐actin and β‐tubulin distribution, suggesting that SIRT2 promotes cytoskeletal reorganization. Conversely, in the SIRT2 knockdown group, F‐actin and β‐tubulin levels were significantly decreased with severe cytoskeletal disruption observed. Notably, in the group co‐expressing SIRT2 and NLRP3, while F‐actin and β‐tubulin levels increased, they were lower than those in the SIRT2 overexpression group, suggesting that NLRP3 overexpression partially counteracts the promoting effect of SIRT2 on cytoskeletal reorganization (Figure 5A,B).

Regarding the expression of cartilage repair‐related proteins, the model group exhibited a significant reduction in COL2A1 and aggrecan levels, alongside a marked increase in matrix metalloproteinase 9 (MMP9) and MMP13, indicating decreased cartilage matrix synthesis and enhanced degradation. In contrast, the SIRT2 overexpression group restored matrix synthesis and inhibited degradation while the SIRT2 knockdown group exacerbated the damage. The co‐expression of SIRT2 and NLRP3 resulted in an intermediate repair capacity, suggesting that NLRP3 partially offsets the beneficial effects of SIRT2 on cartilage repair (Figure 5C,D).

Regarding energy metabolism, ATP level measurements showed a significant increase in the SIRT2 overexpression group (Figure 5E), and electron microscopy revealed an increased number of mitochondria with restored normal morphology (Figure 5F). In contrast, the SIRT2 knockdown group exhibited a marked reduction in ATP levels, a decreased number of mitochondria, and abnormal mitochondrial morphology. In the group co‐overexpressing SIRT2 and NLRP3, energy metabolism results were intermediate between the SIRT2 overexpression and knockdown groups, indicating that NLRP3 overexpression attenuated the protective effects of SIRT2 on energy metabolism.

These findings suggest that SIRT2 plays a critical role in both cytoskeletal reorganization and energy metabolism in chondrocytes by regulating the NF‐κB/NLRP3 axis. Additionally, SIRT2 influences chondrocyte repair and matrix metabolism by modulating key proteins such as COL2A1, aggrecan, MMP9, and MMP13. However, its protective effects are partially diminished when NLRP3 is overexpressed.

### 3.6 SIRT2 promotes cartilage damage repair in a mouse model of OA via the NF‐κB/NLRP3 axis

**FIGURE 6:**
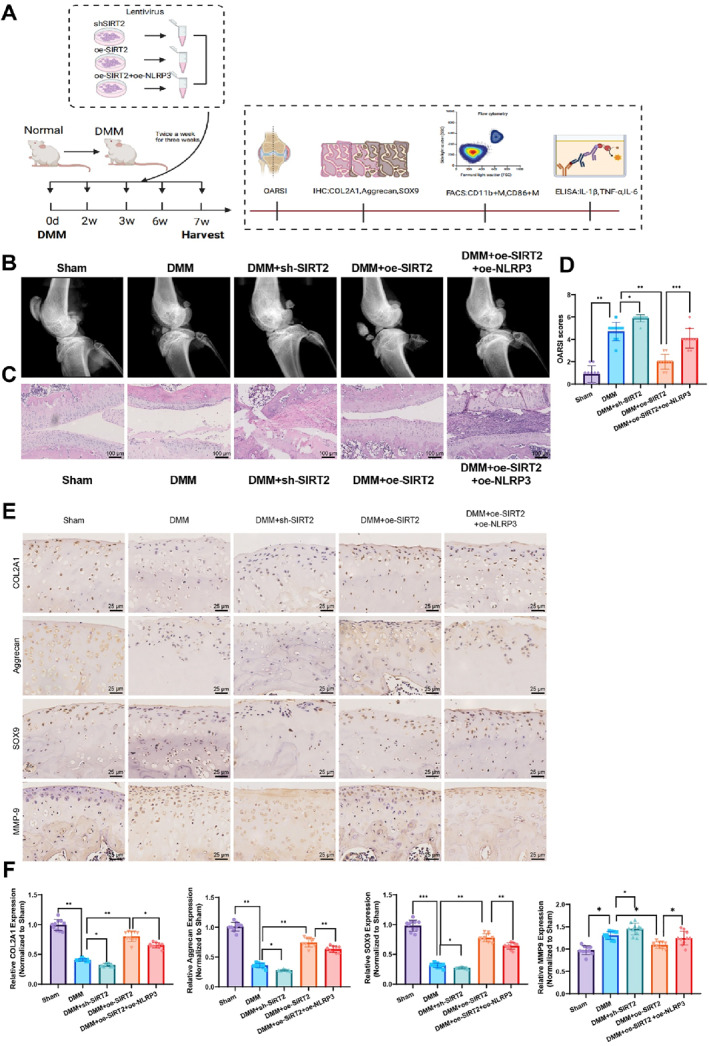
SIRT2 promotes cartilage damage repair in OA by regulating the NF‐κB/NLRP3 axis. (A) Schematic diagram of the in vivo experimental procedure. (B) Assessment of joint cartilage damage through X‐ray imaging, comparing cartilage structural changes among different treatment groups. (C) Histopathological examination of cartilage tissue using H&E staining to evaluate cartilage damage. (D) Osteoarthritis research society international scoring based on pathological changes in joint tissue for the quantitative evaluation of cartilage damage severity in each group. (E) Immunohistochemical (IHC) staining to detect the expression of cartilage repair markers COL2A1, aggrecan, SOX9, and MMP9 in different treatment groups. (F) Quantitative analysis of the relative expression levels of COL2A1, aggrecan, SOX9, and MMP9, comparing differences among treatment groups. All data are presented as mean ± standard error of the mean. The experiments were performed in triplicate with 10 mice per group. Statistical analysis was conducted using analysis of variance and Tukey's post‐hoc test where *p < 0.05, **p < 0.01, and ***p < 0.001. MMP9, matrix metalloproteinase 9; NF‐κB, nuclear factor kappa B; NLRP3, NOD‐like receptor protein 3; SIRT2, sirtuin 2.

In this study, we systematically evaluated the effects of SIRT2 regulation and its interaction with NLRP3 on cartilage damage repair and inflammatory response in a mouse model of OA (Figure 6A). OARSI pathological scoring showed significant improvement in joint cartilage damage in the SIRT2 overexpression group with markedly lower scores compared to the model group. In contrast, the SIRT2 knockdown group exhibited exacerbated cartilage damage, reflected in significantly higher scores than the control group. The rescue group, where both SIRT2 and NLRP3 were co‐overexpressed, showed partial improvement in repair, although not to the same extent as the SIRT2 overexpression group (Figure 6B–D).

Further supporting this trend, IHC analysis of cartilage repair markers confirmed these findings. The expression of COL2A1, aggrecan, and SOX9 was significantly upregulated in the SIRT2 overexpression group, indicating enhanced cartilage repair. In contrast, MMP9 expression was reduced, suggesting suppression of matrix degradation. Conversely, in the SIRT2 knockdown group, the repair markers were downregulated and MMP9 was upregulated, reflecting impaired repair and aggravated matrix breakdown. The expression levels in the rescue group were intermediate between those of the SIRT2 overexpression and knockdown groups, indicating that NLRP3 overexpression partially counteracted the repair‐promoting effects of SIRT2 (Figure 6E,F).

**FIGURE 7:**
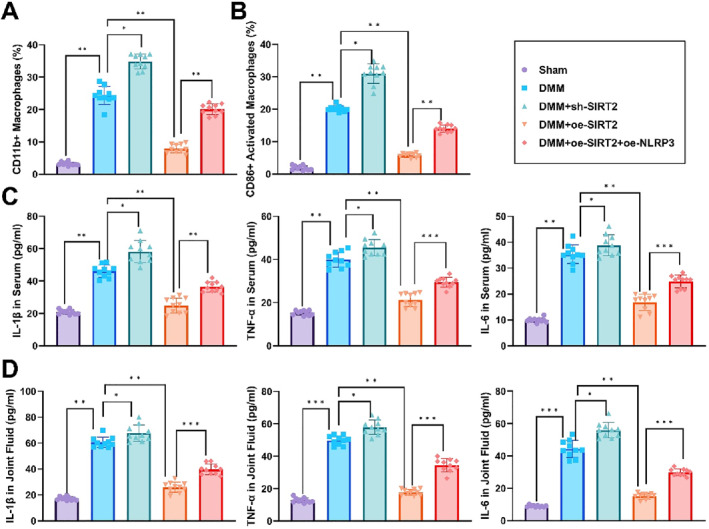
SIRT2 promotes cartilage repair by inhibiting the inflammatory response through regulation of the nuclear factor kappa B/NOD‐like receptor protein 3 axis. (A, B) FCM analysis of the proportions of CD45^+^ leukocytes, CD11b + macrophages, and CD86+ activated macrophages in joint tissues. (C, D) Enzyme‐linked immunosorbent assay results show the levels of pro‐inflammatory cytokines IL‐1β, TNF‐α, and IL‐6 in joint synovial fluid and serum. All data are expressed as mean ± standard error of the mean with experiments repeated three times (*n* = 10 mice per group). Statistical analysis was performed using analysis of variance followed by Tukey's post‐hoc test. *p < 0.05, **p < 0.01, ***p < 0.001.

FCM results further demonstrated that the infiltration of CD45^+^ leukocytes, CD11b^+^ macrophages, and CD86^+^ activated macrophages in joint tissues was significantly reduced in the SIRT2 overexpression group, whereas inflammatory cell infiltration was markedly increased in the SIRT2 knockdown group. The proportion of inflammatory cells in the rescue group was intermediate between the two (Figure 7A,B). ELISA analysis showed that the concentrations of IL‐1β, TNF‐α, and IL‐6 in joint synovial fluid and serum were significantly lower in the SIRT2 overexpression group compared to the model group, whereas these pro‐inflammatory cytokines were significantly elevated in the SIRT2 knockdown group. The levels of inflammatory factors in the rescue group were again intermediate between the two (Figure 7C,D).

These findings suggest that SIRT2 plays a key role in protecting cartilage in the OA mouse model by regulating the NF‐κB/NLRP3 axis, thereby suppressing the inflammatory response and promoting cartilage repair. However, the overexpression of NLRP3 partially counteracted these protective effects.

## 4 DISCUSSION

This study is the first to elucidate in detail the mechanism by which SIRT2 promotes cartilage repair in OA through deacetylation of NF‐κB p65, thereby inhibiting NLRP3 inflammasome activation. This mechanism offers new insights into OA treatment. Although previous research has widely reported the deacetylation function of SIRT2 in various tissues, particularly its role in metabolic and neurological diseases, its function in articular cartilage remains underexplored.^21^ Through both in vivo and in vitro experiments, this study demonstrates the protective effect of SIRT2 on chondrocytes, specifically by inhibiting inflammatory responses and promoting cartilage repair, providing a critical foundation for the development of novel OA treatment strategies.

In contrast to prior studies, this research focuses on the regulatory role of SIRT2 in the NF‐κB/NLRP3 inflammatory pathway. Previous literature identified NF‐κB and NLRP3 as key regulators of inflammation with their overactivation being a major contributor to OA progression.^25^, ^26^, ^27^ However, most studies on NF‐κB have focused on its transcriptional activation with limited attention to the effects of its acetylation on its activity. This study, using co‐IP and ChIP‐qPCR, is the first to clarify the role of SIRT2 in deacetylating NF‐κB, inhibiting its ability to activate the NLRP3 promoter and thus reducing the expression of inflammatory cytokines. This finding provides a new perspective on the regulatory mechanisms of SIRT2 in OA‐related inflammation.

This study also highlights the regulatory role of SIRT2 in chondrocyte proliferation, DNA damage repair, and mitochondrial metabolism. Previous research has established SIRT2 as a critical player in maintaining cellular homeostasis, regulating the cell cycle, and combating oxidative stress; however, these functions have primarily been observed in the liver, nervous system, and kidneys with limited exploration in cartilage tissue.^21^, ^28^, ^29^ Using an in vitro chondrocyte model induced by TNF‐α, this study found that SIRT2 not only suppresses inflammatory responses but also significantly promotes chondrocyte proliferation and repair while inhibiting ROS production and apoptosis. Notably, SIRT2 overexpression greatly enhances mitochondrial energy metabolism, offering new insights into the role of SIRT2 in OA.

The findings from animal experiments further confirmed the crucial role of SIRT2 in repairing OA‐related cartilage damage in vivo. In an OA mouse model, SIRT2 overexpression not only reduced cartilage damage but also markedly decreased inflammatory cell infiltration and cytokine secretion. Particularly, in OARSI scoring and immunohistochemical (IHC) analysis, the SIRT2 overexpression group exhibited significant advantages in cartilage repair, consistent with previous findings on the anti‐inflammatory and reparative roles of SIRT2 in other tissues.^30^, ^31^ Moreover, this study demonstrated that NLRP3 overexpression diminished the protective effects of SIRT2, supporting the conclusion that SIRT2 exerts its chondroprotective effects through NLRP3 inhibition.

In relation to previous clinical studies, this paper highlights the potential of SIRT2 as a therapeutic target for OA. Although earlier research primarily focused on the efficacy of pharmacological treatments for OA, there was limited exploration of modulating endogenous proteins to improve disease progression.^32^, ^33^, ^34^ Our findings demonstrate that SIRT2 not only exhibits potent anti‐inflammatory and cell repair functions in in vitro models but also significantly promotes cartilage repair in in vivo animal models. This study provides a theoretical basis for future clinical trials and may pave the way for the development of novel OA therapies targeting SIRT2.

Despite revealing the critical role of SIRT2 in OA, this study has some limitations. First, the animal model used in this research cannot fully replicate the complex pathological environment of human OA, necessitating future clinical studies to validate the effects of SIRT2 in humans. Additionally, this study only explored the regulatory role of SIRT2 in the NF‐κB/NLRP3 pathway, and its involvement in other inflammation‐related pathways remains unexplored. Future research should employ multi‐omics analyses to investigate the interactions between SIRT2 and other inflammatory pathways. Moreover, the broader biological functions of SIRT2, particularly in various joint tissues, warrant further investigation.

In summary, this study reveals the molecular mechanism by which SIRT2 promotes cartilage repair by deacetylating NF‐κB p65 and inhibiting NLRP3 inflammasome activation. Overexpression of SIRT2 significantly reduces inflammation and enhances cartilage repair, highlighting its potential clinical application. However, the limitations of this study point to new directions for future research such as clinical validation and the exploration of broader signaling pathways. In the future, integrating multi‐omics technologies and conducting in‐depth analyses of SIRT2's function in different joint tissues may offer further insights into potential treatments for OA.

## 5 CONCLUSION

This study clearly demonstrates that SIRT2 promotes the repair of OA cartilage by inhibiting inflammation, oxidative stress, and apoptosis in chondrocytes through deacetylation of NF‐κB p65 which suppresses NLRP3 activation. Animal model experiments showed that SIRT2 overexpression significantly improved cartilage damage in OA mice, reduced inflammatory cell infiltration, and decreased the secretion of pro‐inflammatory cytokines. However, the protective effects of SIRT2 were partially offset by NLRP3 overexpression. Thus, SIRT2 plays a key role in anti‐inflammatory activity and cartilage repair by regulating the NF‐κB/NLRP3 axis, making it a potential therapeutic target for OA (graphic abstract).

While this study highlights the protective role of SIRT2 in OA cartilage repair, further validation of its molecular mechanisms is needed in more complex in vivo and in vitro models. Additionally, the impact of SIRT2 on other types of joint diseases remains unclear, and future clinical studies are required to assess its efficacy and safety. Investigating the interactions between SIRT2 and other signaling pathways is also a crucial focus for future research.

## AUTHOR CONTRIBUTIONS

X.C. and Y.S. conceived and designed the study. F.H., F.Z., and X.C. performed the experiments. Z.D. and J.G. analyzed the data. X.C. and J.G. wrote the manuscript. All authors reviewed and approved the final version of the manuscript.

## CONFLICT OF INTEREST STATEMENT

The authors declare no conflicts of interest.

## ETHICS STATEMENT

All animal experiments were approved by the Animal Ethics Committee of Bengbu Medical University (No. LUNKE PI [2024] No. 196).

## Supporting information

Supporting Information S1

## Data Availability

The data generated or analyzed for this study are available from the corresponding authors upon reasonable request.
