# Supplementary material for: Sirtuin 2 regulates NOD‐like receptor protein 3/nuclear factor kappa B axis to promote cartilage repair in osteoarthritis
Source: J Cell Commun Signal. 2025 Jul 3;19(3):e70031. doi: 10.1002/ccs3.70031 (PMC12226246; doi:10.1002/ccs3.70031)
Supplement: Supplementary file 1 — Supporting Information S1 [file CCS3-19-e70031-s001.docx]

**Table S1. Lentiviral Silencing Sequences.**

| **Name** | **Sequence (5’-3’)** |
| --- | --- |
| sh-NC | CCTAAGGTTAAGTCGCCCTCG |
| sh-SIRT2 | CCTCTATGCAAACCTGGAGAA |

**Table S2. qRT-PCR Primer Sequences.**

| **Gene Name** | **Sequences (5’-3’)** |
| --- | --- |
| NLRP3 **Forward** | GAGTGGACACGAGACAGAGG |
| NLRP3 **Reverse** | TTGCAACGGACACTCGTCAT |
| SIRT2 **Forward** | GAGCCGGACCGATTCAGAC |
| SIRT2 **Reverse** | GCATGTAGCGTGTCACTCCT |
| NF-κB p65 **Forward** | GGATTCCGGGCAGTGACG |
| NF-κB p65 **Reverse** | CACGGCGCGCTAAAGTAAAG |
| IL-1β **Forward** | TGCCACCTTTTGACAGTGATG |
| IL-1β **Reverse** | TGATGTGCTGCTGCGAGATT |
| GAPDH **Forward** | CCCTTAAGAGGGATGCTGCC |
| GAPDH **Reverse** | TACGGCCAAATCCGTTCACA |


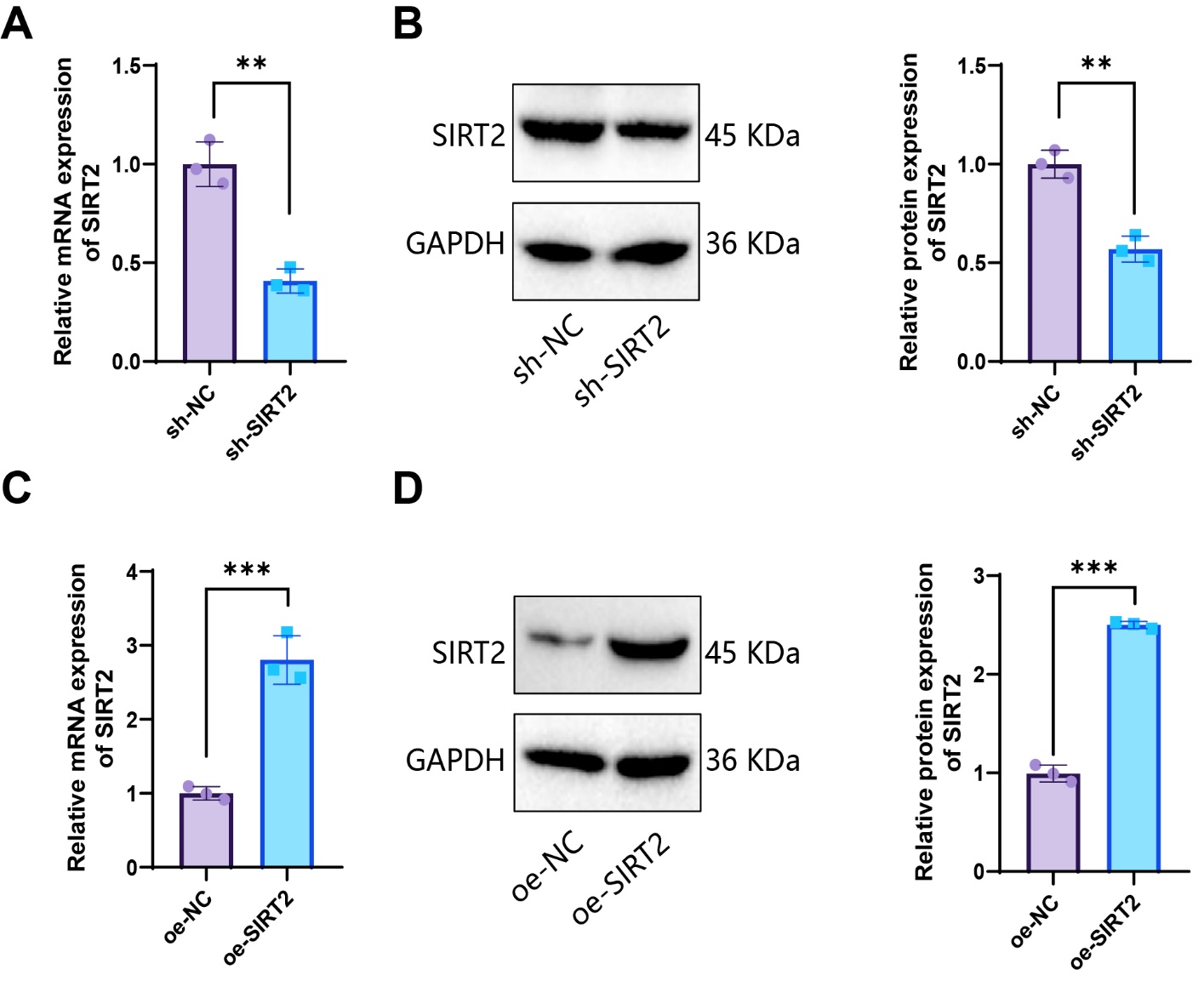


**Figure S1. Validation of SIRT2 Overexpression and Knockdown Efficiency**

Note: (A) RT-qPCR analysis confirming SIRT2 knockdown efficiency using sh-SIRT2. (B) Western blot analysis confirming SIRT2 knockdown at the protein level. (C) RT-qPCR analysis confirming SIRT2 overexpression (oe-SIRT2). (D) Western blot confirming increased SIRT2 protein levels after overexpression. All data are presented as mean ± SEM. Experiments were repeated three times. Statistical analysis was conducted using ANOVA followed by Tukey’s post-hoc test. *p < 0.05, **p < 0.01, ***p < 0.001.


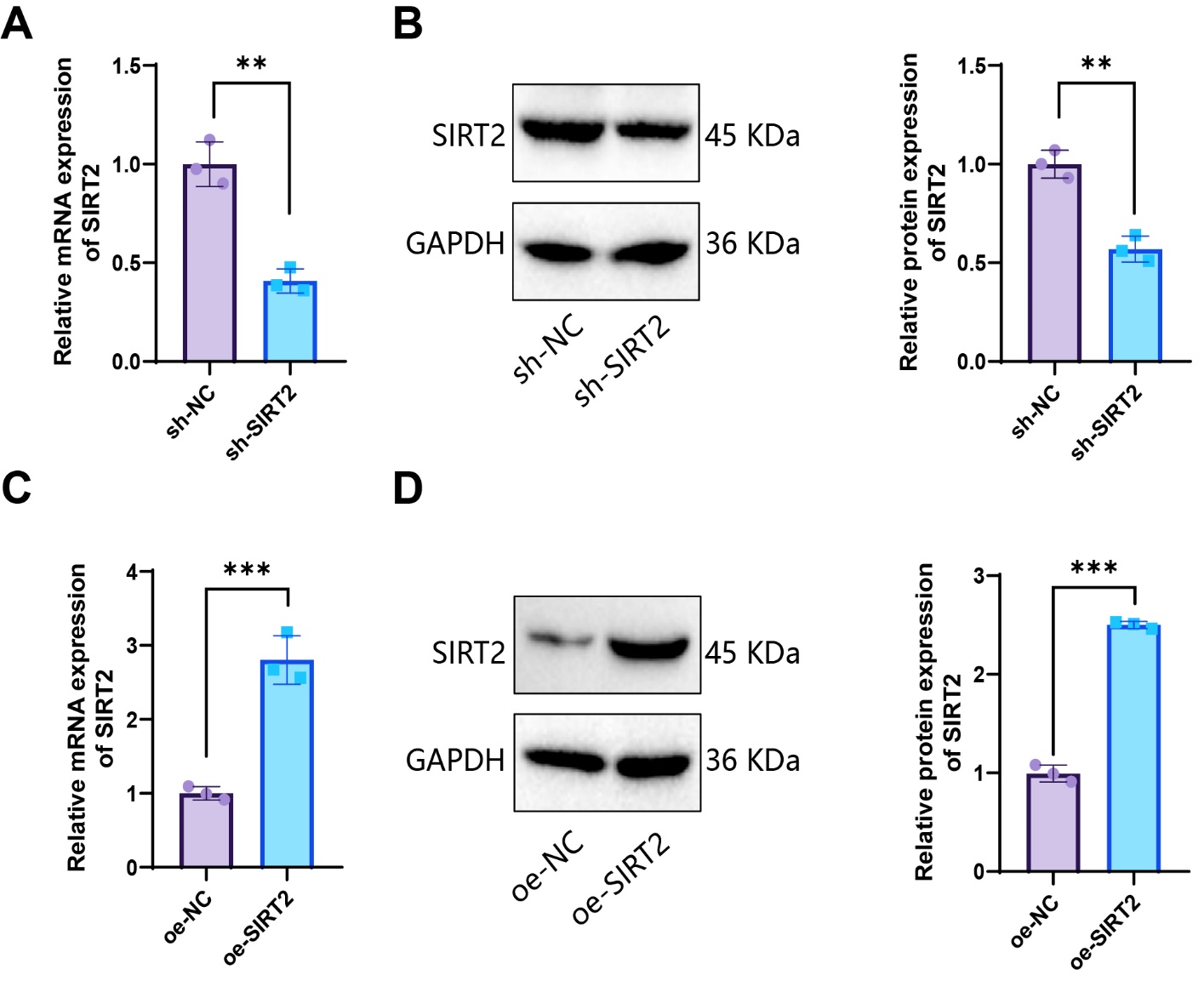


**Figure S2. Experimental Results Showing How SIRT2 Regulates the Cell Cycle and Apoptosis of Chondrocytes Via the NF-κB/NLRP3 Axis.**

Note: (A) Cell cycle analysis displaying the distribution of cells in different phases (G1, S, G2/M) across groups. (B) Apoptosis rate analysis showing the percentage of apoptotic cells in each group, assessed using FCM.
